# Perpetration of physical and sexual abuse and subsequent fathering of pregnancies among a cohort of young South African men: a longitudinal study

**DOI:** 10.1186/1471-2458-14-947

**Published:** 2014-09-12

**Authors:** Nicola J Christofides, Rachel K Jewkes, Kristin L Dunkle, Frances A McCarty, Nwabisa Jama Shai, Mzikazi Nduna, Claire E Sterk

**Affiliations:** School of Public Health, Faculty of Health Sciences, University of Witwatersrand, Johannesburg, South Africa; Rollins School of Public Health, Emory University, Atlanta, GA USA; Gender and Health Research Unit, Medical Research Council, Pretoria, South Africa; Department of Psychology, the University of the Witwatersrand, Johannesburg, South Africa

**Keywords:** Intimate partner violence perpetration, Paternity, Teenage pregnancy, South Africa

## Abstract

**Background:**

Young men’s involvement in fathering pregnancies has been substantially neglected in unintended pregnancy research. Gender norms give men substantial power and control over sexual encounters, suggesting that understanding men’s role is imperative. We tested the hypothesis that young, unmarried South African men who had perpetrated intimate partner violence (IPV) have a greater incidence of fathering pregnancies.

**Methods:**

The data for this study were collected from 983 men aged 15 to 26 who participated in a 2-year community randomized controlled HIV prevention trial in the rural Eastern Cape. Multivariate Poisson models investigated the associations between baseline perpetration of IPV and fathering subsequent pregnancies, while controlling for age, number of sexual partners, socio-economic status, educational attainment, problematic alcohol use, exposure to the intervention, and time between interviews.

**Results:**

Of the men in this study, 16.5% (n = 189) had made a girlfriend pregnant over two years of follow up. In addition, 39.1% had perpetrated physical or sexual intimate partner violence and 24.3% had done so more than once. Men who at baseline had perpetrated IPV in the previous year had an increased incidence of fathering, for a first perpetration in that year IRR 1.67 (95% CI 1.14-2.44) and among those who had also been previously violent, IRR 1.97 (95% CI 1.31-2.94). Those who had ever been violent, but not in the past year, did not have an elevated incidence. The incidence among men who had ever perpetrated physical abuse was less elevated than among those who had perpetrated physical and sexual violence IRR 1.64 (95% CI 1.18-2.29) versus IRR 2.59 (95% CI 1.64-4.10) indicating a dose response.

**Conclusion:**

Young men’s perpetration of partner violence is an important predictor of subsequently fathering a pregnancy. The explanation may lie with South African hegemonic masculinity, which valorizes control of women and displays of heterosexuality and virility, and compromises women’s reproductive choices.

## Background

Recent research has highlighted associations between men’s perpetration of intimate partner violence and several adverse reproductive health outcomes, including sexually transmitted diseases and HIV [1, 2]. There is also evidence that perpetrating gender-based violence is linked to a range of risky sexual behaviors such as transactional sex, higher partner numbers, multiple concurrent sexual partners, and lower condom use, all of which might increase the risk of fathering a pregnancy [3–5]. However, to date only limited research with men has directly explored the question of whether men’s perpetration of intimate partner violence might be associated with fathering of pregnancies, whether wanted, unwanted, or unplanned. One survey of 6632 married men in India found that those who had sexually and/or physically abused their wives were more likely to report an unplanned pregnancy [6]. Similarly, men attending a community health service in Boston who reported perpetrating IPV in the past year were more likely to have fathered three or more children [5]. These limited findings are complemented by a broader body of research with women from a number of different settings, which suggests that women’s experience of intimate partner violence from men is associated with unwanted or unplanned pregnancy [7–9].

Most efforts to prevent unplanned and unwanted pregnancies have focused on women and controlling women’s fertility through contraceptive services. There has been little attention paid to the role of men who father these pregnancies. Men have a role to play in reproductive decision-making and may control whether women use contraceptives or not [10]. In South Africa, women have the potential for greater control over contraceptive use but there are still very potent barriers and one of the more important of these is submission to men’s control either in the face of physical violence or emotional manipulation. Jewkes & Morrell (2012) emphasize the substantial degree to which women voluntarily submit to male control, something which reflects social norms about ‘appropriate’ women’s behaviour, and is to some extent rewarded by male partners [11, 12]. Excluding or limiting men’s involvement in reproductive health programs may limit the potential impact of these programs [10]. Understanding more about the potential role of male violence in predicting unwanted and unplanned pregnancies could help improve prevention efforts and inform multifaceted interventions aimed at preventing both violence and pregnancy.

In light of the health and social consequences for young women, adolescent pregnancy in particular has been a focus in South Africa. Nearly one in five women report having had an adolescent pregnancy, while 5.8% of men report having fathered a pregnancy with an adolescent woman [13]. Risk factors for adolescent pregnancy include lower socio economic status, employment status, having attitudes support sexual permissiveness and contraceptive use [13].

We drew on data from young, rural South African men who participated in the Stepping Stones HIV prevention trial to test the hypothesis that perpetrating intimate partner violence is associated with incident fathering of a pregnancy over approximately 24 months of follow up.

## Methods

South African men aged 15 to 26 (n = 1,368) were recruited from 70 villages near Mthatha in the Eastern Cape, South Africa to participate in a cluster randomized controlled trial of the Stepping Stones HIV prevention intervention [14].

Young women and men interested in participating in the study were invited to a detailed information session where they were encouraged to ask questions. Additionally, each potential participant received a Xhosa-language leaflet describing the study using terms understandable to a lay audience. The leaflet included the phone numbers of staff and toll free helplines [14]. Participants were requested to talk with their families before committing themselves to the study.

The young people who decided to participate in the study were asked to report at an assigned time anywhere from two to seven days after the information session. At that time, each participant provided written consent and study recruitment finalized. Ethical approval for the study and the consent process was given by the University of Pretoria Ethics Committee and the University of Witwatersrand Ethics Committee.

Approximately 20 male volunteers per cluster participated and each cluster received either the Stepping Stones intervention or a short control intervention on HIV prevention. The Stepping Stones trial was conducted between March 2003 and April 2006; results have been published elsewhere [15].

Detailed data were collected on men’s violence perpetration and sexual behavior at each of three time points: baseline (T_0_), first follow-up (T_1_; N = 1,034) which occurred approximately one year after baseline, and second follow-up (T_2_; N = 983) which occurred approximately two years after baseline. One-thousand one-hundred eighty-seven participants (85.3%) had data from at least one follow-up time point and were included in this analysis.

At each time point, face-to-face interviews were carried out by trained male interviewers. Fathering a pregnancy after baseline, the outcome of interest, was measured at both first follow-up and second follow-up with the question “Since the previous interview have you been told by a girlfriend that you made her pregnant?” An affirmative response to this question asked at T_1_ was categorized as fathering an incident pregnancy during the period between the baseline and first follow up and an affirmative response at T_2_ was classified as an incident pregnancy occurring between first and second follow up. To eliminate any misclassification of pregnancies during the follow up period, the dates of when their girlfriend became pregnant and baseline interview dates were compared to ensure that the pregnancy started within the period after the baseline interview and before the first follow up interview and not prior to baseline. Incident T_1_ and T_2_ reports of fathering a pregnancy were combined to create a dichotomous variable that represented having ever or never fathered a pregnancy over the follow-up period.

Perpetration of intimate partner violence by men was measured using an adaptation of the WHO Violence Against Women instrument [16]. This instrument consists of subscales measuring physical abuse (5 items) and sexual violence (4 items) directed towards an intimate partner. An example from the physical abuse subscale is “Since the first interview, did you hit [name of partner] or any other girlfriend with a fist or with something else which could hurt her? Did this happen many times, a few times, once or did it not happen?” An example item from the sexual violence subscale is “Since the first interview did you physically force [name of partner] or any other girlfriend to have sex with you when she did not want to? Did this happen many times, a few times, once or did it not happen?” Men who only responded ‘once’ to all queries about frequency were classified as perpetrating violence only once because even men who endorsed multiple items could have perpetrated only one multifaceted event. All questions were asked both for the past year and ‘before the past year’. Several variables were created to categorize type and intensity of intimate partner violence. A four-level variable classified IPV as no abuse, physical abuse only, sexual abuse only, and physical and sexual abuse perpetrated. Frequency of abuse was a three level variable with no abuse, one episode of physical or sexual abuse, and more than one episode of physical and or sexual abuse. Temporality of abuse was a four level categorical variable with no abuse ever, abuse that ceased before 12 months prior to baseline, abuse that first occurred within the 12 months prior to baseline, and abuse perpetrated both before and within the 12 months prior to baseline (ongoing abuse).

An eight item scale assessed relationship control with a man’s current or most recent partner (alpha = 0.73) [17]. A typical item was “I have more to say than [NAME OF GIRLFRIEND] does about important decisions that affect us”.

Alcohol use was measured using the AUDIT scale [18]. A score of 8 or higher was considered to be indicative of problem drinking. We also asked about use of dagga (cannabis), benzene, mandrax, injected drugs, or other drugs and dichotomized responses into ever and never drug users based on a positive response to having used any one of the listed drugs.

Partner numbers were calculated by summing the responses to questions about the number of main partners, *khwapheni* (hidden partners concurrent with main partners) and casual or “once off” partners reported in the past year. Time since last sex, a rough proxy for coital frequency, was calculated based on the response to the question “When was the last time you had sex?” [19]. Socio-demographic measures included age and completed years of schooling. Socio-economic status was measured on a scale derived for the study encompassing household goods ownership, food scarcity and perceived difficulty accessing a fairly small (but not trivial) sum of money for a medical emergency (R100 which was about $14).

### Statistical analysis

Since the original study was a stratified, two stage survey with villages sampled from predefined strata based on geographical characteristics and participants clustered within villages, initial data analyses were carried out using the survey commands in Stata 10 (Stata Corp., College Station, Texas, USA). These procedures allowed us to account for the lack of independence in the observations (non-zero, positive intra-cluster correlation) because of the sampling design. Descriptive statistics were first calculated for all variables; and two-way associations were determined between categories of IPV perpetration and fathering a pregnancy.

Random effects Poisson models were built to test the hypothesis that baseline perpetration of partner violence and relationship control predicted the incident pregnancies fathered. Four models were built to investigate the association of type of abuse, frequency of abuse, temporality of abuse, and relationship control with fathering. The perpetration of abuse at the baseline (T0) was used as the primary exposure of interest, while the primary outcome was incident fathering of a pregnancy. Each model included variables for the study treatment arm, partner numbers, time since last sex, stratum, and person years of exposure. We tested for interactions between perpetration of IPV and the intervention treatment arm, and perpetration of IPV and substance use, neither were significant. We also assessed the models for confounding by age, education, SES, having a concurrent partner, substance use and duration of primary relationship, and having fathered a pregnancy prior to baseline. Any variable found to affect the point estimate for the main exposure of interest by more than 10% was included in the final model [20]. We tested goodness of fit. We confirmed the findings of associations by modeling survival time under observation using a Weibull model, with the same sets of variables.

## Results

Participants lost to follow-up were compared to those who were retained in the cohort. The 14.7% (n = 205) of men who were lost to follow up were significantly less likely to have ever had a girlfriend at the baseline and those partnered were more controlling (Table 1). There were no other significant differences between those retained in the cohort and those lost to follow up.

**Table 1:** **Comparison of the sample followed up and lost to follow up**

	Followed up n = 1187 (%)	Lost to follow up n = 205 (%)	p value
Age (Mean years)	19.12	19.31	0.17
Education: to grade 10	85.6	88.5	0.16
Socio-economic status (score)	0.009	-0.07	0.51
Ever had a girlfriend	98.1	95.0	0.01
Ever had sex	94.4	92.8	0.32
Fathered a pregnancy	12.8	14.6	0.48
Duration of sexual activity (yrs)	4.76	5.04	0.21
Alcohol problem	25.4	24.3	0.78
Drug use	38.1	40.3	0.59
Lifetime Number of sexual partners			0.73
<=1	10.2	8.3	
2—5	48.6	48.2	
>5	41.2	43.5	
**Relationship power scale:**			0.008
low equity	10.6	19.3	
mid equity	65.8	59.7	
high equity	23.6	21.0	
**Perpetration of IPV against a girlfriend ever by type**			0.29
None	68.6	68.6	
Physical abuse only	22.8	22.1	
Sexual abuse only	3.2	5.1	
Physical & sexual abuse	5.4	3.5	
Intervention: Stepping Stones	51.1	48.1	0.45

The mean baseline age of participants retained in the cohort and included in these analyses was 19.1 years (range 15.2-26.8). While most men (96%) reported having a girlfriend at baseline, none were married at the start of the study or over the two years of follow up. Approximately one in six (16.5%; n = 189) men reported that a girlfriend told him that he made her pregnant over the approximately two years of follow up. The total incidence of known pregnancies was 8.67 per 100 person-years of follow up. The majority of men who reported fathering a pregnancy did not want that pregnancy at all (n = 131, 69.3%) and an additional 25 men (13.2%) did not want the pregnancy at that time.

Nearly a third of men at baseline (31.2%) reported having ever perpetrated sexual and/or physical IPV; 22.6% reported that they had perpetrated physical abuse only, 3.2% had perpetrated sexual abuse only and 5.5% had perpetrated both physical and sexual abuse in their lifetime.

Table 2 shows two-way associations between the demographic and behavioral characteristics of the men at baseline by whether or not they fathered an incident pregnancy over the two years of follow up. Men who reported fathering a child over the follow up period were significantly more likely to report having perpetrated physical and/or sexual violence. They also reported having more sexual partners over their lifetime, were more likely to have had a concurrent sexual partner, and were more likely to have had sex within the past week at the baseline interview. They were also more likely to report problematic alcohol use. Men who reported fathering a child over the follow up period were also more likely to have reported having fathered a pregnancy prior to baseline.

**Table 2:** **Socio-demographic and behavioral characteristics of 1187 young men by reporting fathering an incident pregnancy over approximately 2 years of follow up**

	Fathering an incident pregnancy between T0 & T2 (N = 191)			Not fathering an incident pregnancy between T0 & T2 (N = 996)			P value
Sociodemographic variables	N	%	95% CI	N	%	95% CI	
**Age**							
<18	15	7.9%	4.7-12.8	99	9.9%	7.6-12.8	0.16
18-19	107	56%	47.8-63.9	609	61.1%	57.5-64.7	
>20	69	36.1%	28.8-44.1	288	28.9%	24.7-33.6	
10 or more years of education	87	45.6%	35.8-55.7	437	43.9%	38.4-49.5	0.73
Socio-economic status (score)	-0.002		-0.09-0.08	0.14		-0.07-0.34	0.20
Alcohol problem	63	32.98%	27.0-39.6	238	23.9%	20.6-27.5	0.004
Drug use	84	44.0%	36.5-51.8	368	37.0%	32.8-41.3	0.09
**Sexual and Relationship History**							
Girlfriend in past 12 months	187	97.9%	94.8-99.2	922	95.0%	93.5-96.2	0.06
Lifetime Number of sexual partners							0.002
<=1	45	23.5%	17.8-30.6	309	33.4%	30.3-36.6	
2—5	105	55%	48.0-61.8	499	53.9%	50.7-57.1	
>5	41	21.5%	16.7-27.2	118	12.7%	10.6-15.3	
Concurrent partners (ever)	61.	78%	54.8-68.3	48.60%		45.2-52.0	0.001
Time since last sex <7 days	110	57.6%	48.5-66.3	322	34.8%	30.9-38.9	<0.001
**Fathered a pregnancy prior to baseline**	182	31.9%	25.4-39.3	935	13.1%	10.8-15.8	<0.001
**Perpetration of IPV against a girlfriend by temporality prior to the baseline**							<0.001
None	100	52.9%	45.9-59.8	679	71.9%	68.6-75.1	
Before the past 12 months only	15	7.9%	4.9-12.5	59	6.3%	4.8-8.1	
Within the past 12 months only	38	20.1%	15.0-26.5	128	13.6%	11.4-16.0	
Both before and within the past 12 months	36	19.1%	14.0-25.4	78	8.3%	6.2-10.9	
**Perpetration of IPV against a girlfriend ever by type prior to baseline**							<0.001
None	100	52.9%	45.9-59.8	679	71.9%	68.7-75.1	
Physical abuse only	58	30.7%	23.9-38.4	198	20.8%	18.3-24.0	
Sexual abuse only	6	3.2%	1.4-7.0	30	3.2%	2.2-4.6	
Physical & sexual abuse	25	13.2%	9.2-18.7	37	3.9%	3.0-5.2	
**IPV perpetration by frequency (ever) at baseline**							<0.001
None	100	52.9%	45.9-59.8	690	72.3%	68.9-75.4	
Once only	31	16.4%	11.5-23.0	123	12.9%	11.2-14.8	
More than once	58	30.7%	23.9-38.4	142	14.9%	12.6-17.5	
**Relationship control scale α = .61**	Mean score = 21 (8-29)			20 (11-32)			0.03
Low control	45	24.1%	17.8-31.6	315	34.4%	30.3-38.7	
Medium control	60	32.1%	25.7-39.2	264	28.9%	25.7-32.2	
High control	82	43.9%	36.2-51.9	337	36.8%	32.8-41.0	

Table 3 shows the associations between the type of IPV perpetrated, the temporality of perpetration, the frequency of abuse and incident fathering while controlling for a range of potential confounders including number of sexual partners in the previous 12 months, time since last sex, and study treatment arm. Men who had last perpetrated IPV over a year before the baseline were not significantly more likely to have fathered an incident pregnancy, while men who perpetrated IPV for the first time in the previous year had an increased incidence rate ratio of 1.67 (95% CI 1.14-2.44) and those who had perpetrated IPV both before and within the year prior to the baseline had an increased incidence rate ratio (IRR) of 1.97 (95% CI 1.31-2.94). Men who had ever perpetrated physical abuse only had an increased IRR of 1.64 (95% CI 1.18-2.29) of fathering a pregnancy over the approximately two years of follow up. Men who perpetrated both physical and sexual abuse had an increased IRR of 2.59 (95% CI 1.64-4.10) of fathering an incident pregnancy. Men who had perpetrated one episode of sexual or physical violence had an IRR of 1.56 (95% CI 1.04-2.35) while men who perpetrated more than one episode of physical or sexual abuse in the previous 12 months had an incidence rate ratio of fathering a pregnancy of 1.97 (95% CI 1.57-2.78) demonstrating a dose response. Men who were more controlling in their relationships were also more likely to father an incident pregnancy. Those who were most controlling of their female partners at baseline had an incidence rate ratio of fathering a pregnancy of 1.79 (95% CI1.00-3.29) compared to men who scored highest for equitable relationships. Men who scored in the mid-range for equity in their relationships were also more likely to have fathered a pregnancy (IRR 1.58; 95% CI 1.05-2.37).

**Table 3:** **Poisson model of relative incidence of fathering a pregnancy over the approximately two years of follow-up among young men and having perpetrated intimate partner violence and relationship control at baseline**

**Model 1: Perpetration of IPV against a girlfriend by temporality**				
N = 1133	**IRR**	**95% CI**		**P**
None	ref			
Before the past 12 months only	1.69	0.98	2.93	0.06
Within the past 12 months only	1.67	1.14	2.44	<0.01
Both before and within the past 12 months	1.97	1.31	2.94	<0.01
**Model 2: Perpetration of IPV against a girlfriend by type of IPV**				
N = 1133	**IRR**	**95% CI**		**P**
None	ref			
Physical abuse only	1.64	1.18	2.29	<0.01
Sexual abuse only	1.17	0.51	2.68	0.71
Physical and sexual abuse	2.59	1.64	4.10	<0.01
**Model 3: IPV perpetration by frequency of episodes**				
N = 1144	**IRR**	**95% CI**		**P**
None	ref			
One episode of physical and/or sexual violence only	1.56	1.04	2.35	0.03
Multiple episodes of physical and/or sexual violence	1.97	1.40	2.78	<0.01
**Model 4: Relationship control in current relationship at baseline**				
N = 1103	**IRR**	**95% CI**		**P**
High equity	ref			
Mid equity	1.58	1.05	2.37	0.03
Low equity	1.79	1.00	3.21	0.05

All models control for: number of sexual partners in past 12 months, time since last sex, and treatment arm.

## Discussion

We tested the hypothesis that self-reported perpetration of IPV was associated with subsequently fathering a pregnancy among young men participating in the Stepping Stones HIV prevention trial. We assessed severity of abuse along multiple dimensions including type, frequency, and temporality as well as relationship control – we found that all measures of intimate partner violence perpetration and relationship control predicted fathering an incident pregnancy over two years of follow-up, with more severe perpetration consistently associated with higher likelihood of incident pregnancy, suggesting a dose response. Our results strongly support the hypothesis that young men who perpetrate violence are more likely to father an unwanted or mistimed pregnancy. Our findings complement that body of evidence from cross-sectional studies that show that women who have experienced IPV are more likely to have had an unwanted pregnancy and abortion [21, 22].

Men who had ceased perpetrating IPV before the past year were not statistically more likely than men who had not perpetrated IPV to father an incident pregnancy. Men who had perpetrated IPV for the first time in the past 12 months and especially those who had an established pattern of IPV perpetration were more likely to father a pregnancy over the two years of follow up. This suggests that current abuse and established patterns of abuse are associated with fathering a pregnancy and that the effect may diminish when men have a history of violence perpetration that is not recent.

The suggestion that more severe violence is associated with great likelihood of fathering is informed by the finding that multiple episodes of perpetrating sexual and physical violence had a stronger association with fathering a pregnancy.

Notably, men who perpetrated physical violence only, but not men who perpetrated sexual violence only, were more likely to father new pregnancies. This combined with the finding about relationship control suggests that the association may be more rooted in the broader aspect of power and control in relationships rather than in simple forcing of unprotected sex. Prevention efforts should look beyond sexual violence into the broader context of creating gender equitable relationships.

These findings affirm and extend the small body of literature linking violence perpetration to fathering [6, 23], as well as the literature on violence and pregnancy among women. They are further coherent with literature on male violence perpetration and STD/HIV risk and therefore suggest that recently established links between male violence perpetration and adverse sexual health outcomes include unwanted and unplanned pregnancies.

The findings emphasise the importance of addressing violence in health settings, especially those related to maternal health and abortion. The need to identify women at risk of violence and to engage men and women in interventions to prevent violence during pregnancy and the post-partum has been identified and currently there are a number of studies which are investigating approaches to do so that are both safe and effective [21].

This study is an analysis of data from a cohort of volunteers in an HIV prevention trial, and this may limit the generalizability of the findings. The outcome, fathering a pregnancy, was self-reported and based on when the young men’s girlfriends told them they had made them pregnant. It is possible that some of these reports were erroneous and it is impossible to know with certainty when the pregnancies occurred. This may have resulted in errors in time assessment and either under-reporting or over-reporting of pregnancies. It is possible that female partners who had experienced violence from a man would be less likely to disclose an unwanted pregnancy; however, any such differential under-reporting would have biased our results towards the null, suggesting that point estimates reported may underestimate true effect sizes. The Stepping Stones intervention was focused on transforming gender norms and could have resulted in higher reporting of fathering among the intervention group. We therefore controlled for intervention arm in our analyses. Significant strengths of the study include the use of prospectively collected data, the coherence of our findings across different measures and modeling procedures, and the evidence of a dose-response effect with increasing severity, frequency and recency of violence more strongly associated with fathering.

## Conclusion

We have shown that perpetration of physical and sexual abuse and relationship control by young men in South Africa are associated with later fathering of pregnancies. These findings have implications for both pregnancy prevention and violence-prevention and would suggest that joint programming for preventing gender-based violence and unwanted pregnancies is required. Programs that address inequitable gender power relations among men could result in a reduction of unwanted and unplanned pregnancies among young women.

## References

[1] Jewkes R, Sikweyiya Y, Morrell R, Dunkle K (2009). Understanding Men’s Health and Use of Violence: Interface of Rape and HIV in South Africa.

[2] Silverman JG, Decker MR, Kapur NA, Gupta J, Raj A (2007). Violence against wives, sexual risk and sexually transmitted infection among Bangladeshi men. [see comment]. Sex Transm Infect.

[3] Dunkle KL, Jewkes RK, Nduna M, Levin J, Jama N, Khuzwayo N, Koss MP, Duvvury N (2006). Perpetration of partner violence and HIV risk behaviour among young men in the rural Eastern Cape, South Africa. AIDS.

[4] El-Bassel N, Fontdevila J, Gilbert L, Voisin D, Richman BL, Pitchell P (2001). HIV risks of men in methadone maintenance programs who abuse their intimate partners: a forgotten issue. J Subst Abuse.

[5] Raj A, Reed E, Welles SL, Santana MC, Silverman JG (2008). Intimate partner violence perpetration, risky sexual behavior, and STI/HIV diagnosis among heterosexual African American men. Am J Mens Health.

[6] Martin SL, Kilgallen B, Tsui AO, Maitra K, Singh KK, Kupper LL (1999). Sexual behaviors and reproductive health outcomes: associations with wife abuse in India. JAMA.

[7] Jewkes R, Penn-Kekana L, Levin J, Ratsaka M, Schrieber M (2001). Prevalence of emotional, physical and sexual abuse of women in three South African provinces. S Afr Med J.

[8] Varga CA (2003). How gender roles influence sexual and reproductive health among South African adolescents. Stud Fam Plann.

[9] Pallitto CC, Campbell JC, O’Campo P (2005). Is intimate partner violence associated with unintended pregnancy? a review of the literature. Trauma Violence Abuse.

[10] Gogna M, Binstock G, Fernandez S, Ibarlucía I, Zamberlin N (2008). Adolescent pregnancy in Argentina: evidence-based recommendations for public policies. Reprod Health Matters.

[11] Jewkes R, Morrell R (2012). Sexuality and the limits of agency among South African teenage women: theorising femininities and their connections to HIV risk practises. Soc Sci Med.

[12] Jewkes R, Morrell R (2010). Gender and sexuality: emerging perspectives from the heterosexual epidemic in South Africa and implications for HIV risk and prevention. J Int AIDS Soc.

[13] McHunu G, Peltzer K, Tutshana B, Seutlwadi L (2012). Adolescent pregnancy and associated factors in South African youth. Afr Health Sci.

[14] Jewkes R, Nduna M, Levin J, Jama N, Dunkle K, Khuzwayo N, Koss M, Puren A, Wood K, Duvvury N (2006). A cluster randomized-controlled trial to determine the effectiveness of Stepping Stones in preventing HIV infections and promoting safer sexual behaviour amongst youth in the rural Eastern Cape, South Africa: trial design, methods and baseline findings. Trop Med Int Health.

[15] Jewkes R, Nduna M, Levin J, Jama N, Dunkle K, Puren A, Duvvury N (2008). Impact of stepping stones on incidence of HIV and HSV-2 and sexual behaviour in rural South Africa: cluster randomised controlled trial. [see comment]. BMJ.

[16] Schraiber LB, Latorre Mdo R, Franca I, Segri NJ, D’Oliveira AF (2010). Validity of the WHO VAW study instrument for estimating gender-based violence against women. Rev Saude Publica.

[17] Pulerwitz J, Gortmaker SL, DeJong W (2000). Measuring sexual relationship power in HIV/STD research. Sex Roles.

[18] Saunders JB, Aasland OG, Babor TF, de la Fuente JR, Grant M (1993). Development of the Alcohol Use Disorders Identification Test (AUDIT): WHO Collaborative Project on Early Detection of Persons with Harmful Alcohol Consumption–II. Addiction.

[19] Collumbien M, Gerressu M, Cleland J, Ezzati M, Lopez A, Rodgers A, Murray CJL (2004). Non-use and use of ineffective methods of contraception. Comparative Quantification of Health Risks: Global and Regional Burden of Disease Attributable to Selected Major Risk Factors.

[20] Kleinbaum DG, Klein M (2005). Logistic Regression: A Self-Learning Text.

[21] World Health Organization/London School of Hygiene and Tropical Medicine (2010). Preventing Intimate Partner and Sexual Violence against Women: Taking Action and Generating Evidence.

[22] Pallitto CC, Garcia-Moreno C, Jansen HAFM, Heise L, Ellsberg M, Watts C, W. H. O. Multi-Country Study Women's Hl (2013). Intimate partner violence, abortion, and unintended pregnancy: results from the WHO Multi-country Study on Women’s Health and Domestic Violence. Int J Gynecol Obstet.

[23] Raj A, Santana MC, La Marche A, Amaro H, Cranston K, Silverman JG (2006). Perpetration of intimate partner violence associated with sexual risk behaviors among young adult men. Am J Public Health.

[CR24] The pre-publication history for this paper can be accessed here:http://www.biomedcentral.com/1471-2458/14/947/prepub

